# Inquiring into conditions for engaging in narrative relations on a geriatric ward – how interpretation matters in everyday practices

**DOI:** 10.1080/17482631.2024.2367851

**Published:** 2024-06-13

**Authors:** Lisa Herulf Scholander, Sofia Vikström, Anne-Marie Boström, Staffan Josephsson

**Affiliations:** a Division of Occupational Therapy, Department of Neurobiology, Care Sciences and Society, Karolinska Institutet, Huddinge, Sweden; b R&D Unit, Stockholms Sjukhem Foundation, Stockholm, Sweden; c Division of Nursing, Department of Neurobiology, Care Sciences and Society, Karolinska Institutet, Huddinge, Sweden; d Theme Inflammation and Aging, Karolinska University Hospital, Huddinge, Sweden

**Keywords:** Ethnography, geriatric care, interpretation, narration, person-centred care

## Abstract

Drawing on data from ethnographic fieldwork and interprofessional focus group discussions, this study enquires into staff’s everyday life on a geriatric ward to explore and understand conditions for engaging in narrative relations in in-patient geriatric care. Avoiding individualistic understandings of narrative practices, we applied a narrative-in-action methodology built on a relational understanding of narrativity, where individual narratives are not separated from social and cultural features. This helped us explore how individual interpretations of the conditions for everyday practices come together with broader social or cultural understandings to gain situated insights about how these are continuously related and reformed by one another in everyday situations of geriatric care. The findings offer insights into the opportunities to engage in narrative relations based on how healthcare staff on a geriatric ward interpret conditions for their practices, and how they act based on such interpretations. While some interpretations were associated with attitudes and activities encouraging narrative relations, others simultaneously thwarted narrative relations by enacting task-orientation, division, or a focus on measurable biomedical or function-related outcomes. Moreover, the findings suggest and discuss consequences of the tensions created as interpretations are enacted in everyday healthcare situations, thus questioning assumptions about conditions as something static and linear.

## Introduction

Some things in our mundane lives may easily pass unnoticed while appearing more significant when reflected from the standpoint of other persons involved. At a first glance, a hospital ward may be perceived as a shared place, understood similarly by the people acting and being there. At a closer look, evidently the interpretations of everyday life on the ward vary noticeably depending on whose perspective is taken, while still being constantly communicated in ongoing transactions between people in everyday situations.

In this study we enquired into the space where individual interpretations of the conditions for everyday healthcare activities on the ward come together with broader social or cultural understandings. By being granted access to everyday life on a geriatric ward during ethnographic fieldwork and attending to patients’, staff’s and managers’ reflections on their experiences, we have been able to catch glimpses of everyday healthcare practices and how they are interpreted from different yet communicating perspectives. More specifically, the focus of our inquiry is the evolving conditions and opportunities for engaging in narrative practices on this ward. There is a growing body of knowledge on the use of narration as a resource for person-centred care (PCC) practices. However, both narrative research and research on narrative practices in terms of PCC have traditionally focused on individual-level narratives and narration. In this study we wanted to move away from such individualistic underpinnings by not separating individual narratives from social and cultural features to rather explore situations where individual, social, and cultural level narratives are continuously related and reformed by one another.

## Background

During the last decade there has been a global shift in orientation of healthcare practices towards person-centredness (McCormack & McCance, 2016; Nolte et al., 2020; World Health Organization, 2015). However, the need for integrating different types of knowledge and perspectives to achieve more holistic and humane practices is still challenged by tenacious practices focusing on biomedical aspects, diagnoses and task- and time-orientation (Kitson et al., 2014; Martyushev-Poklad et al., 2022; McCormack et al., 2021). In everyday healthcare work, professionals navigate between different knowledge systems and traditions. They deal with facts and meaning, with biomedical problems but also social, cultural, psychological, and moral issues. Moreover, people or social groups may hold various ideas about the meaning of different phenomena, e.g., healthcare practices, illness, ageing, or what it means being a healthcare professional or a patient, which affects how people respond to those meanings. Everyday tensions between various needs and understandings amongst people, organizations and society prompt meaning-making processes, and giving attention to such processes has been suggested as a promising resource for health and well-being when facing the health challenges of contemporary societies (Knizek et al., 2021).

Narrative forms of reasoning are crucial for healthcare professionals’ meaning-making relating to experiences they deal with in their everyday work and to help create strategies for clinical actions, treatment plans and relationships (Mattingly, 1998). In a contemporary Swedish context, eliciting the patient narrative has been suggested as a central practice for putting the philosophy of PCC into practice (Britten et al., 2017, 2020; Ekman et al., 2011; SBU, 2017). There are also other theoretical frameworks arguing for narrative practices as an essential element in person-centred approaches, which advocate narrative practice not as a technique but an approach or style embedded in all interactions and activities (Buckley et al., 2014; Villar & Westerhof, 2023). Adding the tradition of *narrative medicine* (Charon, 2001) and *life story work*, e.g., (McKeown et al., 2010; McKinney, 2017; Wills & Day, 2008) there are several tendencies pointing at the significance of narrative forms of knowledge in healthcare practices, although the conceptualization of narrative varies from individualistic to relational notions. However, previous research has suggested that in healthcare practice, narrative modes of reasoning as foundation for PCC may often be overshadowed by persistent communication forms aiming to organize information in categories or checklists (Scholander et al., 2021) and by task-oriented approaches (McCormack et al., 2021) where the latter forms are more suitable for biomedical purposes than for supporting relational interactions and meaning-making processes connected to the uncertainties and ambiguities embedded in healthcare practices. So, although narration is fundamental in human meaning making (Polkinghorne, 1988; Ricoeur, 1984), healthcare professionals’ capabilities to explore and interpret the meaning and values embedded in people’s stories may be constrained, due both to individual abilities and organizational reasons. However, there is a risk that a focus on individual narrative competence or individual stories may disregard the relational features of narrative practices, making this responsibility an individual matter.

Several studies have emphasized that a relational perspective on narrative practices hold significance for upholding person-centred practices (Scholander et al., 2021, 2023; Villar & Serrat, 2017; Villar & Westerhof, 2023), thus moving the focus from individual narratives or competencies to instead recognize narration as open-ended co-constructions and ongoing processes of meaning-making. This means a focus shift away from individual competencies to interpret and integrate individual patient stories in healthcare, to a focus more broadly on narration and participation in joint meaning-making processes, including both staff and patients. Moreover, the construction of individual narratives is highly influenced by broader social narratives. Such culturally shared narratives are often normative yet available for people to potentially internalize or resist, either consciously or unconsciously, when constructing their personal narratives (McLean & Syed, 2016).

When acknowledging the relational and collaborative aspects of narrative practices and the transactions between individual narratives and culturally shared narratives, it may be more accurate to recognize and approach narrative competencies on the social level. Here, the concept of *narrative relations* (Scholander et al., 2023) may be a promising resource. The notion of engaging in narrative relations, i.e., narration involving several people in mutual and open-ended processes of meaning making, has been suggested to uphold foundational qualities of healthcare practice. These include building trustful relations, preventing simplistic understandings of people and situations, peer learning and support, as well as supporting continuity and coherence (Scholander et al., 2023). A further area of inquiry is the everyday conditions for staff to engage in narrative relations and how organizational features and dominant narratives are interpreted and influence individual narratives and narrative relations in practice. For instance, conflicting narratives may exist about what activities, organizational strategies or type of information are acknowledged and valued in healthcare work (Scholander et al., 2024). To understand this better, we wanted to explore how individuals are nested within, interpret, and act in relation to, the cultural narratives provided by the organization, different professional traditions, or influential groups in the setting. Hence, we wanted to examine the narratives that influence everyday practice on a geriatric ward to contribute with knowledge about how individual meaning-making, care culture and organization are interlinked in everyday practices of geriatric care, which ultimately affect which activities and practices are possible to engage in.

### Aim and research questions

The aim of the study was to explore and understand conditions for engaging in narrative relations in everyday practices on a geriatric ward. The following research questions have guided our analysis of the data:

- Which conditions are emphasized in staff’s reasoning about how everyday practices evolve?
- How do staff interpret organizational and cultural conditions in relation to narrative relations in their everyday practices?
- How do these interpretations influence staff’s opportunities to engage in narrative relations?

## Methodology and methods

The study was part of a larger project on narrativity in everyday practice of geriatric care, and analyses data generated from ethnographic fieldwork and from interprofessional focus group discussions with healthcare staff. The Swedish Ethical Review Authority approved the study (reference numbers 2019–00248 & 2022–05463–02).

Epistemologically, the study is situated in a constructivist, interpretative tradition. We build on a non-dualistic assumption about a dynamic interplay between individual-level narratives and shared social narratives, and that organizational systems are not only contexts separate from individuals but intertwined through transactional processes (Alsaker et al., 2013). Therefore, when using the term “conditions” for narrative relations in this paper, we do not intend to map out organizational structures or prerequisites effective for supporting narrative relations. Instead, our interest is to explore people’s situated interpretations of conditions that were significant to them in terms of the everyday activities they engage in. Moreover, we assume that narratives can take both verbal and enacted forms, and concern action and experience (Alsaker et al., 2009; Mattingly, 1998; Ricoeur, 1984). Hence, for our purposes of the study we needed to use a methodology that was sensitive to verbal and enacted narratives, as well as individual, social, cultural, and organizational elements, and, crucially, the transactions between them. Thus, we use a narrative-in-action framework (Alsaker et al., 2009, 2013), a methodology that highlights the social and organizational influences on individual constructions of experiences, emphasizing individual-structural transaction, and which offers resources for investigating the processes of negotiating personal and cultural narratives. This interpretive approach helped to go beyond the dualistic notion of individuals and structures by acknowledging the dynamic and ongoing interchange between individual interpretations and pre-existing understandings. An underlying assumption was that individual narratives are bounded within the broader social narratives to which they have access, but not decisively determined by them (Rudman & Aldrich, 2017). This means that individuals can creatively draw upon pre-existing, dominant narratives in their construction of personal narratives, but due to the normative character of culturally shared narratives, individuals are always operating in relation to those.

Moreover, the narrative-in-action methodology acknowledges the connection between narrative and action. Since culturally shared narratives are often invisible and unconsciously integrated in individual narratives, a challenge can be to get participants to intentionally reflect on them in verbal interviews (McLean & Syed, 2016; Syed & McLean, 2023). Employing a narrative-in-action approach (Alsaker et al., 2009, 2013) offered opportunities to obtain insights from ethnographic fieldwork into narratives enacted in everyday action and verbal reasoning, both the dominant narratives and narratives expressed or enacted by individuals to make sense of their situations, actions and experiences.

### Theoretical resources and their application

The narrative-in-action methodology has its theoretical roots in Paul Ricoeur’s philosophy about narrative and action (Alsaker et al., 2009; Ricoeur, 1984) and employs the concept of a threefold mimesis as an analytical resource. Following Ricoeur’s philosophy, the threefold mimesis denotes a notion of narrative interpretation as an ongoing process instead of mimesis as an imitation, thus offering a resource for exploring possible understandings rather than fixed representations (Josephsson et al., 2006, 2022; Ricoeur, 1984). Thus, the meaning of a narrative is not assumed to represent a subject or person *behind* the narrative, but meaning is constructed *from* the narrative in a forward-thinking interpretation (Kristensson Uggla, 1994). Moreover, the interconnection between narrative and action that Ricoeur derives is central in our use of this theory (Josephsson et al., 2022; Ricoeur, 1984).

Any composition and interpretation of a narrative is based on a preunderstanding of the world of action and the meaning of actions, referred to by Ricoeur (1984) as mimesis_1_. Action, if understood as something that someone does, is always embedded in meanings; actions have motives explaining why someone does or did something, and they are based on previous experiences and preunderstandings. Thus, actions are embedded in the past, the present and the future, and there is not only one possible meaning or motive of a specific action. Hence, they call for interpretation. We have found the Ricoeur’s notion of a threefold mimesis (Alsaker et al., 2009; Josephsson et al., 2022; Ricoeur, 1984) useful as an analytical resource since the three folds of mimesis reflect that individual experiences and meanings are pre-configured by social influences such as dominant narratives as acknowledged by transactional perspectives, yet not locked to those dominant narratives. The notion of a threefold mimesis process helps make visible the interpretations that give rise to everyday actions. This process is not linear but an iterative movement between the mimesis folds. While mimesis_1_ refers to cultural preunderstandings of action or practice that may be unconscious or not yet verbalized, mimesis_2_ refers to the emplotment, where actions and elements are configured into possible, conscious plots (Alsaker et al., 2009; Josephsson et al., 2022; Ricoeur, 1984). However, the mimetic process will not be realized without communication and reception of the narrative, which is what mimesis_3_ refers to. The reception of a narrative, whether by a reader, a spectator or oneself presents an opportunity for interpreting the narrative from other possible preunderstandings, thus opening towards re-configuration of understandings in turn offering opportunities to redefine preunderstandings and mimesis_1_. This shows how mimesis is not a stepwise process from one to three, but how the mimetic activities are intertwined in a continuous process.

Understanding narration in healthcare as a transactional phenomenon implies that one cannot separate individuals and their narratives from the environment and culturally shared narratives, as these continually communicate (Alsaker et al., 2013; Rudman & Aldrich, 2017). Consequently, we understand narrative relations as part of the environment; the multiplicity of narratives in every social context involves interchange and mutual influence through the web of narrative relations. Therefore, rather than focusing on narratives as products or results, we will focus on the narrative activity as processes. However, narrative relations are not neutral in the sense that all narratives have the same impact on the culturally shared understandings; dominant narratives and hierarchies may favour certain understandings over others.

### Data generation and analysis

We draw mainly on two types of data material; fieldnotes comprising just over 72,700 words from ethnographic fieldwork conducted by the first author on an in-patient geriatric ward in March-June 2019. The fieldwork included participant observations and informal conversations with patients, their relatives, and staff (Hammersley & Atkinson, 2007; Thomson, 2011). Written material gathered on the ward, such as descriptions of care processes, work procedures, guidelines and information brochures directed to patients were mainly used in a preparatory phase before initiating participant observation. We also generated data through seven interprofessional focus group discussions with staff on the ward between May 2020 and August 2021. The transcripts comprise 87,800 words. The purpose of the focus groups was to gather participants to jointly discuss conditions and experiences of everyday healthcare practice (Denzin & Lincoln, 2018) in relation to the themes PCC and narration, to explore individual and shared experiences through the group interaction (Morgan & Spanish, 1984). We used vignettes portraying multilayered everyday situations on a geriatric ward to prompt discussions, and follow-up questions to deepen the discussions and keep them on topic.

Together, the two data sets encompass ample descriptions of the care environment, of organizational and cultural aspects, and of people’s situated actions in relation to these. This is important in terms of credibility according to our epistemological position in interpretive constructivism: we view interpretations as situated and constructed. Hence, rather than reasoning in terms of data saturation, this requires eventful data that includes rich description of context, multiple examples supporting the presented findings and absence of data that contradict the findings (Josephsson & Alsaker, 2015).

In terms of analysis, Polkinghorne (1995) make a useful distinction between narrative analysis and analysis of narratives; this study pertains to the first. This mean that the data need not offer complete narratives with a beginning, middle and end, but may also include shorter narrative fragments as, and descriptions of, events and situations, which through the interpretative analysis process are configured into plots (Josephsson & Alsaker, 2015). In the analysis we focused on the ongoing narrative interpretations embedded in participants’ reasoning or actions, using the three mimesis folds as an analytic resource to bring the dimensions of narrative interpretations together. This helped us move beyond merely mapping out conditions and prerequisites for practice, to explore the transactions between pre-existing culturally shared understandings of conditions (mimesis_1_), individual narrative interpretations made towards these preunderstandings (mimesis_2_), and the communication and refiguration between them (mimesis_3_) (Alsaker et al., 2013). This hermeneutic process involved articulating tentative interpretations relevant to the study aim while constantly reverting to the data to check whether those interpretations were supported or not (Gustavsson, 2000). This included reading the data several times, noticing recurrent themes related to the aim and research questions, and articulating interpretations, while constantly returning to the research questions; hence, a hermeneutical process of moving back and forth between data, emerging interpretive ideas and emplotments until the final interpretations were judged reasonable, and satisfactorily supported by the data. The process also meant exploring tensions and contradictions between the conditions that were emphasized by the participants, and which emerged through analysis as significantly affecting the everyday actions that they incited.

### Setting

The study site where we generated data was an inpatient geriatric ward at a Swedish hospital in the Stockholm area. The ward had a 42-bed capacity, over three floors. Reasons for admittance could be rehabilitation needs after emergency treatment or recently declining health situations requiring interventions from a multidisciplinary team with competence in geriatric healthcare. The multidisciplinary team included nurses, nursing assistants, senior physicians with specialist competence in geriatric medicine, junior physicians, occupational therapists, physiotherapists, a speech and language therapist and a dietitian. One nurse and one nursing assistant had full-time roles as care coordinators. On average, the length of stay was 7.8 days, ranging from a few days up to a month due to the variety of patient needs. The ward had no formal age limits for admission but reported patient aged 64 and older. The most common reasons for admittance were heart disease, infections, fractures, and obstructive pulmonary disease. During the period we conducted the focus groups, one floor was at times dedicated to only COVID-19 care. Around 15% of patients were diagnosed with dementia, although according to staff experience the frequency of cognitive impairment among the patients was higher. Multimorbidity was also common.

There was a deliberate attempt on this ward to encourage different professions to work more closely together. Hence, all professions shared the same office spaces, and participated in team rounds together. However, physicians, nursing staff and allied health professionals also had daily uni-professional meetings.

Staff turnover among nurses and junior physicians was high. Around 70% of nurses had 0–2 years of professional experience and junior physicians had often graduated recently and not started their residencies. Staffing was more stable regarding nursing assistants, allied health professionals and chief physicians.

## Findings

### Overview

**Figure 1.**
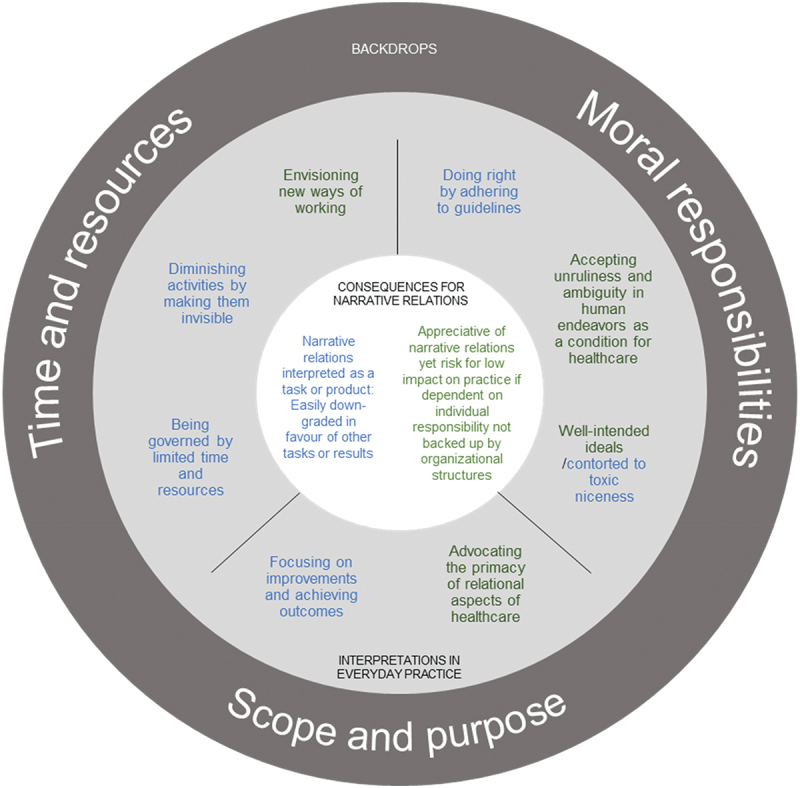
Overview of the findings. The colours indicate contradicting interpretations within the same backdrop and the core consequences they withhold.

The report of the findings is organized according to three different, yet interconnected, layers (see Figure 1) responding to the three research questions. The findings portray how multiple layers of interpretation of the everyday conditions affect which healthcare practices are established, and how that may influence engagement in narrative relations.

The outer layer represents continually reflected preunderstandings about key conditions considered influential for everyday healthcare practice, which we have chosen to call backdrops. The second layer represents various, yet common, interpretations made towards the three different backdrops. In other words, this implies that various meanings were ascribed to the backdrops. The third layer, portrayed in the last section of the findings, represents the possible consequences for narrative relations.

### Backdrops

The backdrops, *Moral responsibilities, Scope and purpose of healthcare*, and *Time and resources*, were not fixed preconditions but subjects for interpretation, yet they seemed substantial and commonly referred to, thus affecting the kind of practices that were possible to realize. The backdrops were not predefined but emerged through the analysis as participants discussed, responded to, and negotiated such backdrops in action, and our analysis showed diverse interpretations rendering different meanings from them. Hence, backdrops may encompass an array of interpretations, while these interpretations transactionally contribute to form the meaning of the backdrops. As we will show, the various interpretations made from each backdrop were sometimes in conflict with each other, thus their parallel existence created tensions in everyday activities and interactions.

In the following sections we present each backdrop (layer 1) by portraying the various interpretations (layer 2) relating to it, to show how these interpretations influence activities and priorities of everyday healthcare work on the ward and, directly or indirectly, influence people’s engagement in narrative relations (layer 3). Along with the interpretations we will give situated descriptions and examples from the data, or present excerpts from field notes and quotes from the focus groups to illustrate our findings. Subsequently, we will discuss an example showing how conflicting narrative interpretations may be played out in everyday practice.

### Backdrop: moral responsibilities in healthcare

#### Doing right by adhering to guidelines and routines

Common, yet rarely explicit, was a reliance on guidelines and pre-defined routines, aiming to safeguard patient safety and ensure correct action was taken. To-do lists were a frequently used tool, both observed in practice and recurrently reflected in discussions between participants. Several documents described the different professional groups’ work processes and responsibilities on quite a detailed and task-oriented level. For instance, certain questions should be posed to patients on admission, certain instruments should be used for predicting risks common among patients on the ward, measures should be taken for safeguarding good nutrition, elimination or preventing falls, and medicines should be distributed at the right time. In terms of communication, the guidelines stated: “communicate by following SBAR”,^1^ which often created fact-oriented conversations and thwarted engagement in narrative relations. The reliance on guidelines and routines seemed aligned with an underlying idea made visible through what some participants emphasized as significant and what they left out when talking about their responsibilities in practice:

The chief physician sits down with me for a moment and tells me about geriatrics and the characteristics of that field. She says there are so many things to keep track of: nutrition, risk of falls, cardiovascular function, mental well-being, cognitive function, bowels, neurology, pain and so on. I notice she mainly talks about biomedical aspects, not mentioning anything about the social or existential. “One has to consider everything”, she repeats. Excerpt from fieldnotes

Sometimes practices enacted from this preunderstanding indicated convictions of one superior way of doing things; by adhering to that, the right and good action was safeguarded. Whilst adequate for some purposes, this idea sometimes seemed to spill over to other, relational, aspects of practice; e.g., adhering to guidelines and predefined procedures could contribute to a sense of security and predictability among staff, patients, and relatives. Admitting a single action as the right measure prevented engaging in uncomfortable practices of dealing with uncertainties or admitting lack of knowledge. For instance, it was frequently considered the right action to refer the patient to someone else when not having the answer to a patient’s question. Although adequate for the type of questions with a clear, factual answer from an expert, this appeared problematic when applied to patients’ existential issues or in their quest for making sense of their situation. In everyday practice, this was made visible through ethical dilemmas such as when patients rejected the action prescribed by the guidelines, which opened for staff to reflect on conflicting moral ideals regarding what is right and good. Thus, tension between ideals were not only negative, but offered opportunities for engaging in narrative relations. However, arenas for taking advantage of such opportunities were scarce. When efforts were made to establish such arenas, for instance daily checkout reflection meetings, they often relied on checklist-based formats prompting short answers instead of narrative reasoning. Hence, the moral of doing right according to guidelines did not seem adequate for encouraging engagement in narrative relations, since that type of practice may be better aligned with other morals.

Another example portrays the conflict between everyday circumstances and the belief in adhering to recommendations for practice. In every patient room there was a whiteboard beside each bed where physiotherapists or occupational therapists could write about the patient’s needs relating to assistance with mobilization, re-positioning or activities of daily living (ADL). The conclusions written on the board were often clearly communicated to the patient and written in agreement based on the assessment made. However, we noticed that nursing assistants often expressed frustration with the recommendations as they did not always agree with the assessment made by the rehabilitation staff, or they felt insecure in how to assist the patient safely without risk of injuring themselves or the patient. Nevertheless, the practice of whiteboard recommendations was seldom questioned. Rehabilitation staff, on the other hand, reported frustration with low compliance by nursing assistants to the recommendations, obstructing possibilities for rehabilitation embedded in everyday activities. This example aims to portray how the simplest facts or information are not straight-forward but situated, interpreted, and affected by those involved in a particular situation, which we will illustrate next.

#### Accepting unruliness and ambiguity as a condition for everyday work in healthcare

Another interpretation relating to the backdrop *Moral responsibilities* indicated a recognition of ambiguity, contradictions, and unruliness as inevitable conditions for healthcare practices. Staff, including several different professions, often acknowledged that it was hardly ever possible to stick to the envisioned plan, as unexpected events continuously occurred, expressed as a basic condition for this kind of work. This view often seemed connected to accrediting processes as much as outcomes, ascribing intrinsic value to actions and responses in the present moment rather than solely focusing on improving medical or functional outcomes, which at times was not even feasible when dealing with complex health issues and the stipulated time limits on the ward. Our analysis indicated that when staff accounted for, or acted from, this understanding, they seemed more often appreciative and supportive towards engaging in narrative relations. However, such convictions were not necessarily robust or frequent enough to manifest in everyday practice when competing with other sturdier social constructions, e.g., interpretations of the meaning and consequences of limited time, or task-oriented routines.

Moreover, this understanding acknowledged that people may have contradictory interpretations based on their different vantage points, which was seen as a basic condition for human interaction. Consequently, there was an acceptance of interpretation as a necessity for understanding and relating to other people, but also to formalized guidelines and routines, which cannot be followed literally but which need to be interpreted and applied in relation to individual situations.

While waiting for the nurse and nursing assistant to arrive at the team rounds, the chief physician, junior physician, physiotherapist, and occupational therapist engage in a discussion about their struggle with a patient’s son considered to meddle too much in the care of his father. Family members’ opinions may not always lead to the best decisions for the care, they conclude. According to the chief physician, the son in this case seems knowledgeable of the guidelines, yet he does not understand that guidelines do not state in black and white how to be interpreted in different situations. “There is always room for interpretation, but he seems to be a legalist, becoming upset when not having it his way,” she says. “I talked to him for 45 minutes this Friday, and after that the physio talked to him for an hour.”

Excerpt from fieldnotes

Since this unruliness may clash with other understandings, such as the moral of strictly adhering to guidelines, it would require time and space in healthcare practice for negotiations between staff, patients, and family members to facilitate reaching common ground. As implied in the excerpt above, such discussions may claim time and resources, whether such resources have been deliberately allocated or not.

#### Well-intended ideals contorted to toxic niceness

I think, if they had recognized the patient’s worry earlier and done it in a better way, it wouldn’t have escalated into that situation. But that attitude she [the nurse] has, saying that some patients are quite demanding … perhaps she hasn’t done such a good job really, but she doesn’t want to admit it, so there may be some scope there [laughter].

Nurse, focus group 1

Junior physician: There is a high personal responsibility assigned to the different professions to work magic and to have a perfect attitude every day. I guess that if you are told to mind your attitude, it doesn’t matter, because then you go to your shift being as stressed as you were the day before … struggling to keep up with your workday while you just want to go away and cry because it is just too much. Then it is terribly hard to take on this demand, this stress of conscience, that you are expected to smile all the time [little laughter].Nursing assistant: I must not yawn. [laughter]Junior physician: Certainly not so the patient notices it.

Dialogue, focus group 6

Yet another influential interpretation from the analysis relating to the backdrop *Moral responsibilities* displayed double messages relating to virtues making people suitable for a job in healthcare, and also ambiguous consequences on narrative relations. This interpretation clearly builds on a well-intended moral of doing good, being respectful and appreciating relational aspects of care. However, in practice things seemed more complicated because such expectations also construct individualistic, idealized images about characteristics that make people *“right”* for working in healthcare. Through the analysis we identified an often-indicated disapproval regarding certain behaviours where staff failed to be there for the patient, or to talk about patients in what was considered right and respectful terms, accusing individuals of a lack of interest in the patient or of having the wrong attitude towards the job. When widening the lens from assessing individual action to interpreting the same action in relation to organizational situations observed or discussed, such actions seemed in many cases quite comprehensible. The analysis showed how staff felt expected to not let their feelings show, and let their personal needs come second. While this might be adequate and necessary at times, it becomes problematic if this kind of emotional work is not acknowledged or if resources to recover from such emotional strain are scarce. This may prevent staff from expressing frustration over situations due to risks of judgemental reprimands, whether from others or internalized, thus clearly contrasting to the culture of engaging in narrative relations in practice.

### Backdrop: scope and purpose of healthcare practice

#### Advocating the primacy of relational aspects of healthcare

As seen in the previous backdrop, one common interpretation emerging from how staff reflect on their work, assigns ethical and relational aspects of healthcare practices as the most important qualities, thus affecting the envisioned scope of practice. According to this interpretation, the processes of engaging and interacting with other people have an intrinsic value, particularly emphasized in relation to the commonly existing needs of the patient group on the ward. Consequently, adequate resources were requested for staff to be able to engage in relational practices, including both organizational opportunities to engage with others and resources ensuring that staff’s bandwidth to engage in relations-building practices.

According to this understanding, healthcare practice is ultimately about nurturing relationships, which includes taking time to listening to the patients’ stories, always remembering and acknowledging who the person is in their wider life context. Following this understanding, acknowledging people where they are and supporting them in making sense of their current situation has intrinsic value. Such practices were not always easy for staff in relation to other competing ideas about the scope and purposes of practice.

I think that part has lapsed. That you forget for what reasons you are here. Why you are working with this. You must not forget that this patient, who may be 75 years old now, once was … like … a pilot, or whatever, but fully capable of making their own decisions. And he is still capable of that, but he might need some extra … some extra help. Do you see what I mean? So … don’t forget who they were before … well, you must never forget that. It is so easy to do that.

Nursing assistant, focus group 2

Also, this scope and purpose of practice involved engaging in daily reflection together with colleagues and listening to their stories from everyday practice, acknowledging different perspectives depending on professional affiliation or different experiences. The consequence of this interpretation on narrative relations related mainly to the status of engaging in narrative relations, implying an appreciation of relation-building practices as essential to healthcare work, which must be prioritized both embedded in everyday interactions and given enough margins by the organization. However, such practices were not always enacted in practice or given sufficient room in the organization of everyday work. For instance, we identified a frequently expressed desire for mentoring or other activities for collegial support and joint reflection, but also for sustainable practices of reasonable time for staff to take breaks and recover. The intentions envisioned with such measures were to help staff manage the distress of everyday practice, develop professionally and personally, and strengthen their own resources to be able to engage in the relational aspects of healthcare work. Such activities may offer arenas for engaging in narrative relations but were mostly considered as insufficiently established in this setting, although efforts had been made to try out various activities. A lack of such supportive resources was often reported as a risk for undermining staff’s capabilities of living up to the ideals of being there for patients and for their colleagues.

Well, it is more like I should be able to … I would like to sit on the sofa with a cup of tea, and drink it for ten minutes or something, and then I can move on, because such micro pauses matter a great deal. At least for me they affect how I respond to patients, because I do not as easily get irritated, and everything gets better in general … Psychologically better for me …

Nursing assistant, focus group 6

Interpretations that give intrinsic value to human relations may be enacted in activities and actions aimed at building relationships with other people, whether patients, their next of kin, or colleagues. Hence, engaging in narrative relations seemed aligned with such interpretations regarding the scope of practice.

#### Focusing on outcomes and tangible improvements in medical or functional status

From the analysis, a frequent preunderstanding that influenced activities on the ward emerged, suggesting that measurable outcomes or improvements in medical or functional status were the objectives of being admitted onto the ward. Hence, based on this interpretation, working towards certain outcomes was what ascribed meaning to the activities, and when improvements were impossible to achieve, the stay on the ward was therefore sometimes perceived as meaningless both to staff and to patients. Due to restricted time limits, acting from this interpretation often resulted in a narrow scope, focusing on the condition used as reason for admittance onto the ward, although several participants also problematized this and witnessed moral distress from being pushed to limit their treatment of a diagnosis rather than a person, particularly when working with the comprehensive needs characteristic to the patient group on this ward.

Well, I think time can be troublesome, especially with geriatric patients, because they may be admitted for one reason but then you discover a lot more that is wrong, and then it is often like “yeah but now we are focusing on the reason for admittance, because if we treat that problem they will be satisfied”, and then you are caught between wanting more time and being able to focus on several problems that would need treatment. But you’ve got to … yeah … discuss that in the team, deciding to “yes but no, now we go for this. The other things can be followed up by outpatient services”.

Junior physician, focus group 4

This medical and outcome-oriented scope of healthcare often endorsed a task-oriented focus, promoting adherence to guidelines and stipulated procedures as the leading moral principle. Here, narrative relations were assigned low status, and possibly even considered outside the scope of healthcare practice. An implication interpreted from this narrative may be that healthcare is best organized by linear processes and procedures, exposing an implicit logic of predictability, where specific assignments are expected to lead to the desired outcome, whether it be stabilizing a certain medical value, adjusting medication, or improving limitations in activity or participation. The dominance of such aspects seemed endorsed by a strong and common acceptance of the importance of written documentation, whereby this type of information is favoured at the expense of more complex reasoning which may be impossible or undesirable to record. Interpretations related to this dictated that record-keeping was the most objective and reliable way of making information available to others; it thus motivated time-consuming and high engagement in writing and reading records, instead of face-to-face verbal interaction. However, as those conclusions regarding objectivity and reliability may apply to hard facts, records could not claim to capture the meaning of those facts, which rather were reached through interpretative processes. Moreover, records and written information best captured static facts, thus offering low value in capturing or supporting ongoing processes of meaning-making, relationship building or the transactional aspects of interacting with patients or colleagues, which affects the outcomes, as exemplified previously in terms of the bedside whiteboard with static facts about patients’ needs for assistance in moving or ADL. Hence, the idealistic idea of the superiority of written information was challenged as our analysis showed how written information was only partial and, for some purposes, overestimated, thus giving a false sense of security about seeing the full picture, and risked discarding engagement in narrative relations.

### Backdrop: time and resources

#### Being governed by limited time and resources

A frequently occurring understanding concerned how a lack of time and resources greatly influenced what kind of practice it was possible to uphold. However, this understanding included different interpretations, where separate professions were pressured by different sorts of time limits. Our analysis implied that nurses and nursing assistants in general felt most pressured to keep up with their basic tasks and responsibilities on a daily basis, not having time for taking proper breaks, nor living up to the moral commitments of caring practices, causing emotional and moral distress. Although an assessment of patient care needs was taken into consideration on admittance, narrow timeframes did not always allow for unexpected occurrences, such as when staffing resources were allocated to double manning or keeping continual watch over a patient, and someone else had to take over their tasks. Physicians and allied health professionals instead referred to feeling pressured by restricted timeframes for patients’ length of stay on the ward, where these were decided even before the individual patients’ needs are identified, making meaningful goals hard to achieve, leaving both staff and patients feeling dissatisfied and frustrated. The unit managers offered a third interpretation relating to limited time; they attributed the problem of staff working overtime to staff’s high ambitions and difficulties prioritizing and restricting themselves to a reasonable workload. This understanding implied the lack of time and resources in healthcare was less a structural matter but related more to individual professionals. However, managers also pointed out a problem overheard from employees that staff working the subsequent shift had expressed discontent when the preceding shift left them with unfinished tasks.

Limited resources could also include under-dimensioned physical areas for staff to perform their duties; a frequent example, showing how physical space might both restrict and encourage narrative relations, related to the medical dispensary. This room was a confined space, where often three nurses worked simultaneously, sometimes also supervising students. Around the room, shelves and cupboards were filled with medical packets and material, and three portable working stations in a row made it hard to move around effortlessly. Nurses often grumbled about this, yet they also often discussed the intimacy and support emerging from working so closely together in a place that was only accessible to them and no other profession, unless they let them in. However, due to the small space they also avoided being there at the same time, if possible, thus reducing informal togetherness. The same contradiction was seen in the shared office where narrative relations seemed supported by the physical proximity to others, yet not everyone had access to a computer, making some people chose to perform administrative tasks elsewhere, and mostly meet with others at meetings often ruled by a more formal agenda.

Overall, following this interpretation, little time was left to engage in narrative relations. Overall, it implied that people were pushed to spend their days handling emergencies instead of engaging in moral and relational aspects, which participants in general emphasized as the truly important part of healthcare practice. Simplistically, this interpretation took for granted a certain organizational structure and working procedures based on tasks that must be finished before the shift end, or before the patient is to be discharged after a stipulated length of time, which is mostly regarded as too short for the ward’s patient group. Moreover, when taking a certain way of working for granted, this interpretation did not consider other possible ways of allocating tasks in the team, prioritizing or valuing different kinds of actions or objectives. When the most urgent matters obliviously become the most important matters by being given priority in action, low-key aspects that never called for immediate attention were always set aside. Relational and ethical aspects and activities that promoted cultural development towards such practices seemed most highly valued in participants accounts of what is important in healthcare work, as previously described. Yet, in their everyday work staff often felt pressured to abandon such activities, giving the narrative about lack of time and resources as an explanation. If this interpretation conveyed the full picture, the solution would be more time and better staffing. However, when contrasted to other narratives, a more complex picture appeared.

#### Diminishing activities by making them invisible

They [the junior physicians] tell me that after reading up the records it is time for the team rounds, then the “walking round” when they meet with the patients. I tell them I would like to join them for both these activities, which they agree to.

“I’m only intending to stay until noon,” I add.

“But before that is when most things happen anyway,” one of the physicians says, turning towards her colleague looking for confirmation. Her colleague nods.

“In the afternoon it is more like this,” she says, referring to the gesture of twiddling her thumbs.

Excerpt from fieldnotes

A frequently encountered interpretation relating to *Time and resources* was portraying “times when nothing happens”, opposing the narrative about the constant shortage of time to complete necessary tasks where staff are always stressed. This interpretation created a clash between how activities were labelled and valued by participants, i.e., “nothing happens”, while in the actions observed, several activities took place. Such activities included cleaning up the dispensary room or re-arranging flowers to create a pleasant environment, having coffee with a patient or informal chitchatting with colleagues blended with work-related information and narrative exchange related to work issues. Hence, situations offering opportunities for taking care of relations and well-being on the ward, including engaging in narrative relations, seemed lowly valued or not even noticed. According to the interpretations built from the premise of scarce time and resources, engaging in narrative relations had not a high priority, which seemed grounded in an understanding of narration as a distinctive task, something that should be conducted in addition to and separate from other tasks. Our analysis indicated that narrative forms of knowledge and skills typically seemed to be under-valued in this way. A senior physician on the ward once said: “Previously in geriatric care we had more time to listen to patient stories. Now, that is not the case anymore” (*fieldnotes*). The loss of such reductions was reflected by a woman who had been admitted as a patient on the ward several times.

’Just look at me now! It means so much that you come back and ask me how I am doing, telling me that you are interested in continuing our conversation from yesterday. But there is no time for that. They [the staff] do not take time for that.’ She tells me that it is easy to just end up slouching in one’s chair, disappearing inside oneself. When I addressed her, she says, she came to life, illustrating this by pulling herself up to a more upright posture, turning towards me with open arms. She stresses that this is very important. Excerpt from fieldnotes

Additionally, diminishing relational activities consequently also signalled that such activities, including the abilities and knowledge forms they demand, were of less value.

Other poignant examples of this were the recurring, yet hidden, debriefing in the dispensary room (*fieldnotes, focus group 5*) where the nurses described how they surreptitiously told each other about things that mattered to them, exchanged strategies for how to handle certain work-related dilemmas, or just vented their sometimes-overwhelming feelings—a narrative way of reasoning. Other professions described how they sometimes needed to let off steam or discuss relational matters that were not covered by the team round structures, in the corridors. That these daily needs were not met in more systematic ways and carried out stealthily, signals that these aspects of care work were systematically overlooked.

#### Envisioning new ways of working

Although rarer, an alternative interpretation relating to time and resources was discerned through the analysis, opposing the narrative about lack of time but also bringing new aspects to the backdrop *Scope and purpose of healthcare*. This narrative suggested that time could be saved through new ways of working. For instance, the managers questioned the often-enacted idea that “everything had to be done before the team rounds or before lunch” (focus group 7). They talked about how they tried to encourage staff to question why everything had to be completed as fast as possible instead of redistributing some activities to the usually less hectic afternoons when both shifts overlapped. For instance, some people may not wish to wake up early and eat breakfast, and exploring such personal habits among the patients could be useful to help spacing out activities over the day to the benefit to both patients and staff.

Another identified example was a narrative about the importance of a strong team to balance unevenly distributed demands of practice and help to relieve individual professionals from too much strain. Since this narrative also offers a clarifying illustration of the everyday complexity when conflicting interpretations ascribed to the same narrative unfold in practice, we will discuss this narrative more thoroughly in the next section.

Purposefully using the physical premises to support interprofessional collaboration was another example of how work on the ward was striving towards new, relational practices. Purposefully reducing physical boundaries between professional groups, such as separate offices or meeting rooms, aimed for closer interprofessional cooperation, where it was easier to find physical arenas for engaging in narrative relations.

### Consequences for narrative relations in everyday practice—how tensions between interpretations matter

Moving towards the centre of the illustration in Figure 1, the following section discusses examples of how tensions between interpretations of everyday conditions may affect what kind of healthcare practices people engage in, including opportunities to engage in narrative relations.

As seen in previous sections, there was a recurrent tendency that some narrative interpretations, often expressed verbally and repeatedly, concealed enacted narratives, thus exposing a discrepancy between what was going on in practice and the moral ideals that were not necessarily enacted or established in practice. From here on, we will introduce the term *concealing narratives* to demarcate narratives that were frequently presented about practice yet seemed compromised when compared to the activities and actions taking place. Concealing narratives affirmed the existence of a phenomenon, while an immanent vagueness in fact created a false sense of that phenomenon. They are important to consider in relation to the everyday conditions for healthcare work in general, yet here we discuss the conditions for engaging in narrative relations in particular: firstly, if concealing narratives remain unnoticed, they may contribute to preservation of practices no longer desired and to obstruct change towards new practices and cultures. This may be relevant in terms of accepting change towards person-centred cultures supportive of narrative relations. Secondly, consciously allowing for reflection on taken-for-granted narratives may open an arena for negotiating and challenging existing narratives, which we in turn understand as the realm of moving from seeing narratives as static products to narrative relations as processes.

The narrative about the strong team is one example of a concealing narrative emerging through our analysis. While participants generally affirmed a strong and supportive team on the ward, we observed a vagueness in who was included in the “we” or “us” or “our team”, and what actions were suggested to construct that sense of social cohesion. Sometimes, only a certain professional group was intended, affirming intra-professional cohesion and cooperation, while at other times all professionals on the ward were included. Although this vagueness of everyday speech may seem innocent, concealing narratives created veils by affirming situations that were not really accurate or reflected in practice, thus obscuring what was really going on. Expressing and reflecting on such narratives among participants sometimes led to a refiguration of their understandings where their first interpretations stood out in relief when contrasted with enacted narratives. Displaying a seemingly well-functional situation, while in fact the actual circumstances were hidden, may stand in the way of change, causing good intentions to be lost, yet offering ambiguous explanations of why that happened. A closer look at the narrative about the strong team in turn exposed two opposing narratives, a narrative about *sameness* and a narrative about *diversity and inequalities*.

The narrative about sameness declared that all staff on the ward were working towards a common goal, they had shared interests, and everybody was helping one another. Accordingly, everyone was there for all patients, and covered for each other when necessary. This narrative contributed to concealing the fact that different professions seemed to have quite different everyday conditions for how to manage their practice. Some examples of everyday conditions that generally vary between professions were: different expectations on professional groups to be present at the ward at all times, where nurses and nursing assistants had less opportunity to leave the ward; different opportunities to manage their own time, where nurses and nursing assistants had to respond to other’s immediate needs, requiring them to constantly re-schedule, while physicians and allied health staff felt more in charge of their schedule; different types of tasks, ranging from mostly administrative duties to relational work with patients; or different views of what counts as work or breaks, where allied health staff and physicians more often reported that they intentionally tried to not discuss work during breaks, while nursing assistants often discussed work-related matters during breaks. This is expressed in a narrative interpretation about diversity and inequalities, acknowledging a perspectivist understanding of being and acting on the ward. This alternative narrative shows the fragility of the narrative about sameness and the seemingly shallow notion that everyone working on the ward shared the same foundation and conditions. According to this interpretation, roles, professional affiliation, education, and personal experiences strongly influenced people’s perspectives, yet constantly interplayed with, and were shaped by, the dominant narratives on the ward. Different professional groups seemed prone to act from different dominant preunderstandings in their everyday practice, although this does not mean that generalizations can be made for all individuals belonging to a certain group, since the processes on narrative interpretation differ due to individual preunderstandings. Depending on one’s position, the conditions and expectations were disparate. On one hand, there was a conscious and agreed upon allocation of tasks and responsibilities; on the other hand, the consequences of such divisions were not immediately recognized. A focus on profession-specific tasks disregarded the common features shared by healthcare professionals irrespective of professional affiliation, creating a responsibility gap where such less defined obligations risked becoming omitted. Interplaying with the narrative interpretation about the constant lack of time, this task-orientation pushed people to create firm boundaries around their respective responsibilities, to protect themselves from being overburdened by endless demands. Building walls around oneself further reinforced division and isolation, instead of appreciating the shared aspects and responsibilities of healthcare work. Focusing on protecting boundaries made it harder to reach out for help but also to see things from other people’s perspectives and gain insights into each other’s everyday situations. This risked creating a downward spiral where narrative relations were impeded, thus furthering isolation and individualist perspectives, in turn furthering obstruction of narrative relations.

Being unaware of the differences may result in interpreting other people’s actions from one’s own position. Here, concealing narratives risked creating a false sense of common agreement, while in fact understanding of others was less than realized. Our analysis generated several examples where inadequate or one-sided understanding of the actual circumstances of everyday practice made good intentions fall short, as solutions to problems build on premature and partial insights into the situation. For instance, ambitions to create an arena for daily interprofessional reflection on the day’s work and cooperation, hence likely promoting narrative relations, were appreciated by most professionals, as shown in the backdrop *Moral responsibilities*. However, in practice such initiatives gradually diminished into nothing, and divergent reasons for this were suggested by participants, showing how the actual work conditions for different professional groups and organizational routines had not been fully understood.

Another example is the dual notions about the existence of hierarchies; some people on the ward strongly impugned the existence of hierarchies, while others acknowledged that traditional hierarchies were still affecting the care culture significantly, contributed to ascribing different values to different professional perspectives, and prevented interprofessional cooperation. Narratives about hierarchies as non-existent, or at least a waning thing of the past, risk being concealing, thus hiding the consequences of remaining hierarchies and the interpretive prerogatives, alliances of loyalty or possible conflicts they may entail. This may be related to the fact that dominant narratives more easily become visible for people who are in opposition to them, while remaining invisible to people who align with them, or benefit from them.

The narratives about sameness versus diversity and inequalities may also be discussed regarding person-centredness, including all people on the ward, not only staff. Although a division between patients and professional roles may remain, the division may cause challenges to partnerships if staff fail to remember that patients’ perspectives on being on the ward may completely differ from their own, while still sharing the sameness of being people. For staff, being on the ward was part of everyday life and work. For patients in many cases, being on the ward and facing illness may be perceived as a disruption to their everyday lives. This highlights the ethical responsibilities, as originally argued by Frank (1995), to engage with patient stories and validate their efforts to create new meaning around their situation when facing illness. However, staff were continuously challenged to negotiate patients’ wishes and personal preferences to make them fit within the organizational frames as well as with their own needs. One example from our analysis shows a situation where under-dimensioned resources in the clinic backstage made staff fulfil their unmet needs of recovery and connection with others at the expense of the patients, by using social areas intended for patients for themselves, while patients were excluded from those areas, although undoubtedly not by ill will from the staff. Our analysis also shows how task-orientation and being pressed to hastily perform a chain of routines on the one hand fosters simplistic views of patients where the person is forgotten, and on the other hand creates a gap between areas of responsibility where shared responsibilities, such as responding to patients requests for information or engaging in narrative relations, were left unattended. Oblivion about discrepancies in perspective may seem easy to remedy, yet our analysis shows that it may sneak up on people even though they subscribe ideologically and morally to person-centred values. Moreover, the culture of division where staff were pushed to look after their own responsibilities and tasks may be hidden by concealing narratives about helpful attitudes towards each other. No doubt, generosity and supportive attitudes between staff on this ward were observed and often verbally encouraged; however, in practice, those virtues were repeatedly obstructed as people often lacked bandwidth to provide timely assistance beyond their own remits, being thus counter-productive to adopting a culture supportive of narrative relations.

## Discussion

The aim of this study was to better understand the conditions for engaging in narrative relations in everyday practices on a geriatric ward. More specifically, we were interested in exploring how staff on the ward interpreted the conditions for their everyday practice, and how these interpretations may influence the opportunities to engage in narrative relations. The impetus to explore this comes from previous research suggesting that practices of engaging in narrative relations in geriatric care may contribute to uphold foundational qualities of healthcare practices such as building trustful relations, preventing simplistic understandings of people and situations, promoting peer learning and support as well as supporting continuity and coherence (Scholander et al., 2023)—qualities congruent with features of person-centred practices (McCormack & McCance, 2016). The focus on narrative relations meant a broadened scope beyond merely focusing on routines of eliciting patient narratives, towards narration involving several people in mutual and ongoing processes of meaning-making based on the assumption that individual narratives cannot be separated from broader social and cultural narratives. Instead, those narratives are understood as continuously related and reformed by one another. Hence, the study was less concerned with mapping out and describing organizational structures or processes than understanding the meanings people assign to various conditions of everyday work and the consequences of those meanings when influencing everyday actions. As argued by Knizek et al. (2021), facilitating meaning-making processes in healthcare may contribute to individuals’ health and well-being, yet has not received sufficiently attention. This study is an attempt to contribute to the topic. By emphasizing interpretation and meaning-making in everyday life on a geriatric ward, the findings show how a multitude of meanings ascribed to conditions for everyday practices affects what kind of practices are enacted and upheld.

One contribution of our findings is that they illuminate how people give significance to various organizational factors, values, and preunderstandings, here called backdrops, and how their interpretations and actions are made in ongoing communication with such backdrops. Using the term backdrops instead of conditions to organize the findings signifies a deliberate attempt to distinguish ideas of conditions as something static and linear towards a more dynamic understanding of conditions as something that may not be given, but which instead offer material for ongoing interpretation that may contribute to reinforce or resist the conditions’ influence on everyday practices. Although our analysis showed that participants generally seemed to agree that the backdrops influenced what practices were possible, it simultaneously became clear that these backdrops were not fixed, and did not mean the same thing for all people and over time. In other words, the term backdrops corresponds to the notion that everyday conditions or structures are not independent of human interpretation, but are continuously upheld and reformed by human interpretation and action (Alsaker et al., 2009, 2013). Previous research has suggested several organizational and professional elements, prerequisites, and processes to consider when aiming for transforming practices and care cultures towards person-centredness (Bokhour et al., 2018; Cardiff et al., 2018; McCormack & McCance, 2016). While, for instance, leadership, supportive organizational systems or physical environment might also be considered important conditions for practices of narrative relations yet not reflected as backdrops, it might be worthwhile to reflect on whether such conditions can also be understood as backdrops in accordance with what our findings indicate, thus requiring engagement with how they are interpreted and enacted in everyday situations. In the findings, conditions such as prescribed moral responsibilities of staff, expected scope and purpose of the healthcare on the ward, and the available time and resources were often reflected by participants as determining how practices turned out. When viewed as backdrops, these conditions are rather understood as generally recognized circumstances given particular attention in people’s meaning-making around their everyday practices, thus also affecting their actions. Overall, the analysis indicated how everyday practices on the ward presented a multitude of parallel and sometimes conflicting interpretations of what different organizational elements meant in practice, and consequently, conflicting conditions in terms of opportunities to engage in narrative relations. Our findings imply that all the backdrops could prompt both interpretations, aligning with narrative relations and interpretations antagonizing engagement in narrative relations, consequently creating tension in everyday practices. These tensions contributed to inhibit change towards the desired and intended relational practices. Through analysis we identified several examples of good intentions falling short when managers and staff made deliberate attempts to create opportunities for interprofessional cooperation and reflection. Since such relational practices may offer an important opportunity for fostering narrative relations, these tensions inhibiting relational practices are an important finding. It directs the attention towards such tensions per se, and raises questions about how to intentionally notice and handle them productively.

Moreover, our findings indicated that the activities observed on the ward were sometimes contradictory to the moral ideals that most people adhered to when reflecting on the values connected to their practices. This discordance between most participants’ acknowledgement of certain values and the focus of the activities systematically taking place on the ward is worthy of reflection. If people affirm the existence of a practice that perhaps does not really exist, it might simultaneously contribute to inhibit change towards that desired practice. We suggested the term *concealing narratives* to label this phenomenon of generally accepted, often verbally reinforced, narratives inconsistent with the narratives expressed in action made visible through the analysis. When acknowledging only one narrative of what is deemed good or right in a local context, other narratives may be disregarded while still being enacted in practice. Two examples of concealing narratives from our analysis were a narrative about sameness and one about non-existing hierarchies, hiding practices still influenced by hierarchies and inequalities between professional groups. Analysis indicated that such division between groups may obstruct narrative relations and create fragmentation on the ward, which has been more thoroughly discussed in a previous paper (Scholander et al., 2024). If staff from different professional groups presuppose a narrative of sameness, thus assuming that they interpret the conditions for their everyday practices in the same way, even though that might not be the case, there is little need for creating arenas in everyday practices where tensions between people’s understandings could be explored and negotiated, which seemed to be a barrier for fostering cultures of narrative relations, including interprofessional learning. Instead, fully accepting the narrative about diversity and inequalities would have ethical implications for how to bring the different perspectives forward and not systematically prioritize certain interpretations over others. This raises questions about who benefits from preserving dominant narratives that determine what is prioritized on the ward and what is systematically disregarded. Without any arena to make such parallel narratives communicate, there is a risk that narratives are understood as fixed or locked positions, instead of ongoing processes where narratives are continuously evolving in communication with other narratives. Moreover, this makes visible a potentially problematic function of narratives; concealing narratives become a veil that affirms a desired situation while not accurately portraying what is really going on. This has ethical implications and calls for ongoing reflection regarding whether the narratives broadly presented really reflect what is going on in practice. In turn, this may offer possibilities to use the tensions and discordances productively, and intentionally admit and challenge practices that are no longer desired. Also, some resources such as staff’s appreciative mindsets about relational aspects of healthcare work seem to be widespread but somehow kept locked. Finding ways to unlock those resources instead of aiming to implement something new may also be a productive way forward, that needs to be better understood. Although more research is needed to develop tools supporting such practices, existing research on reflective practices and workplace learning among healthcare professionals may offer synergistic insights (e.g., Lane & Roberts, 2022; McHugh et al., 2020; Mertens et al., 2018)., Moreover, as our analysis did not cluster subgroups and therefore cannot make any claims about identifying profession-specific understandings, research exploring possible differences between subgroups may also provide useful insights regarding divergent interpretation of the conditions for everyday practices. Such knowledge would be valuable in terms of obtaining a better understanding of organizational culture in healthcare, as cultural change has been argued to be what person-centredness is ultimately about (McCormack & McCance, 2016; McCormack et al., 2021), which also might be accurate in terms of fostering narrative relations. Schein’s (1985) often-used definition of organizational culture states that it is “the pattern of shared basic assumptions—invented, discovered, or developed by a given group as it learns to cope with its problems of external adaptation and internal integration—that has worked well enough to be considered valid and, therefore, to be taught to new members as the correct way to perceive, think, and feel in relation to those problems”. However, our findings related to the narrative about diversity and inequalities raise questions related to the existence of multiple organizational subcultures acting on the ward and if the assumptions of some subgroups are systematically privileged over others. As suggested by Clark (2015), different professional groups are socialized to see healthcare practice through unique, profession-specific lenses. Hence, subgroups may hold various basic assumptions that obstruct reaching common ground among all staff on a ward.

More broadly, our findings contribute to portraying the tensions occurring in everyday practices when traditional biomedical and task-oriented cultures are challenged by the morals promoting biopsychosocial, relational, and person-centred practices widely acknowledged in the contemporary healthcare discourse (Kitson et al., 2014; McCormack et al., 2021). Moreover, this is significant in relation to the recognized notion of moral distress among healthcare workers when pressed by institutional constraints to act against one’s moral judgements (Jameton, 1984; Morley et al., 2019; Oh & Gastmans, 2015), and raises questions about how best to support health professionals in their everyday practices when they are implicitly or explicitly expected to navigate between multiple, and sometimes contradictory, missions and principles.

### Methodological considerations

Using ethnographic data enabled our inquiry into how context-specific ideas, values and norms shaped people’s understandings and practices (Hammersley & Atkinson, 2007), and provided insights into the everyday practices of healthcare professionals (Thomson, 2011), while the narrative-in-action framework allowed for bringing together individual, social and cultural aspects in the analysis, thus expanding the analytic focus beyond individual experiences (Alsaker et al., 2009, 2013). Drawing on data from both participant observations and focus group discussion, along with chosen theoretical resources, it was possible to obtain situated insights into the continuous communication between culturally shared narratives and individual narrative interpretations affecting the everyday actions on a geriatric ward. Moreover, the methods made it possible to contrast what was commonly expressed verbally among staff on a specific site with what was commonly taking place in action on the same site. Although participants themselves did not conceptualize their practice in terms of narrative relations, the rich empirical material enabled contextualized interpretation regarding the opportunities to engage in narrative relations on this ward. However, the methodological choices and proceedings are not without limitations, which must be addressed. Whilst the two complementary data sets and the polyphonic nature of the data may offer insights into both individual narratives and dominant narratives influencing the culture on this ward, the reflections made in the focus groups are not immediately connected to specific individual actions, hence, the verbal reflections refer to common practices on the ward more broadly. This means that our interpretations of these data must be understood as analytical abstractions, and we do not make any claims to explain underlying motives or individual meaning tied to specific actions observed. The narrative-in-action methodology (Alsaker et al., 2009, 2013) supports our understanding of the continuous transactions between individual and culturally shared narratives, at the same time admitting the challenge to distinguish individual narratives from shared understandings. Hence, we do not make any claims on rendering all possible interpretations and backdrops guiding the participants’ actions. For instance, one may argue that other aspects such as physical environment or profession-specific narratives could be understood as backdrops. As this may be true, our purpose in the way of presenting the findings was foremost to show how parallel interpretations born from the same backdrop influence everyday practices and the opportunities to engage in narrative relations, instead of providing a complete chart of the conditions for narrative relations in everyday practices. Another limitation is that we did not analyse other sources accounting for the formal organizational structures, processes, financial aspects, or mission statements, which was beyond the accessible resources for this study. However, better understanding of how care cultures are influenced by organizational or societal constructions and discourses merits more research.

Finally, the notion of narrative relations is still an emerging concept only beginning to unravel. While the study was an explorative attempt to generate insights regarding narrative relations in the context of everyday practices and to start identifying what practices and conditions influence opportunities to engage in narrative relations, we believe that the situated insights our analysis demonstrates are valuable for better understanding everyday healthcare practices and the challenges presented when aiming to change persistent healthcare cultures towards more relational and person-centred practices. However, the still quite abstract and immeasurable notion of narrative relations contributes to a vagueness in the findings. Hence, more research is needed to advance our understanding of how narrative relations in everyday healthcare practices can be further developed together with staff in their practices, and how such practices can be evaluated.

## Concluding remarks

This study is an explorative attempt to better understand the conditions for narrative relations in the everyday practices on an in-patient geriatric ward. Our analysis contributes with important insights into the dynamics of the conditions for everyday practices on a geriatric ward and shifts the focus from conditions as something unambiguous and static, to conditions as backdrops to the ongoing interpretation that healthcare staff engage in while carrying out their activities. This means that conditions are understood as continuously interpreted, upheld, resisted, or reformed by the everyday practices people engage in. The backdrops emerged through analysis as commonly referred to conditions significant for what kind of practices were considered possible to realize and included the moral responsibilities in healthcare, scope, and purpose of healthcare practices, as well as available time and resources. The findings show how multiple interpretations were made towards these backdrops, creating tensions in everyday practices, where some interpretations favoured relational practices beneficial for narrative relations, while other interpretations reinforced traditional biomedical, task-and-time-oriented practices less constructive for fostering narrative relations. Although relational aspects of healthcare work were often emphasized by participants as a moral responsibility and even considered as the scope and purpose of healthcare practices, this was sometimes accepted as a concealing narrative that contributed to hide the fact that those values were idealistic and not really enacted in the everyday practices. With this follows an ethical implication for healthcare organizations to create arenas in everyday practice where healthcare professionals are continuously supported to reflect on practices in relation to the values that are commonly affirmed.

## Data Availability

The data that support the findings of this study are available on reasonable request from the corresponding author. The data are not publicly available due to privacy or ethical restrictions.

## Note

1. SBAR (situation, background, assessment, recommendation) is a technique to improve communication and ensure patient safety (Haig et al., 2006).
